# MALT1 in cerebrospinal fluid: a prognostic biomarker and potential therapeutic target in Alzheimer’s disease

**DOI:** 10.3389/fneur.2025.1732729

**Published:** 2026-01-06

**Authors:** Siyi Jiang, Ping Qi, Jia Tian, Huaizheng Liu, Chuanzheng Sun

**Affiliations:** 1 Department of Emergency, The Third Xiangya Hospital, Central South University, Changsha, Hunan, China; 2 Postdoctoral Station of Clinical Medicine, The Third Xiangya Hospital, Central South University, Changsha, China; 3 Department of Burns and Plastic Surgery, Affiliated Hospital of Zunyi Medical University, Zunyi, Guizhou, China; 4 Montana College of Osteopathic Medicine, Rocky Vista University, Billings, MT, United States

**Keywords:** Alzheimer’s disease, biomarker, cerebrospinal fluid metabolites, immune regulation, MALT1, Mendelian randomization

## Abstract

**Background:**

Alzheimer’s disease (AD) is a devastating neurodegenerative disorder, and early intervention remains the only reliable strategy to slow its progression. Notably, cerebrospinal fluid (CSF) metabolites play a crucial role in the early diagnosis of AD, making their investigation highly significant.

**Methods:**

We integrated two-sample Mendelian randomization (MR), transcriptomic, and machine learning (ML) analyses to identify causal CSF metabolites and their downstream molecular mediators in AD. MR assessed the causal effects of 338 CSF metabolites on AD risk, while integrated GEO datasets (GSE4757, GSE48350, and GSE122063) were analyzed to identify 50 differentially expressed associated genes (DEAGs). Immune infiltration and correlation analyses were performed to characterize immune infiltration. Predictive ML models, including Random Forest (RF), Support Vector Machine (SVM), Generalized Linear Model (GLM), and Extreme Gradient Boosting (XGBoost), were used to screen biomarkers, construct a diagnostic nomogram, and validate findings *in vivo*.

**Results:**

The MR analysis identified 15 potential CSF metabolites associated with AD. Elevated creatine levels (OR = 0.610, 95% CI: 0.441–0.845, *p* = 0.003) were protective against AD, whereas increased leucine levels (OR = 1.548, 95% CI: 1.210–1.981, *p* < 0.001) were associated with higher AD risk. Transcriptomic analysis revealed 50 DEAGs enriched in lipid metabolism, inflammation, and neural signaling pathways. Immune infiltration analysis demonstrated altered adaptive and innate immune populations, linking risk genes to immune activation. ML analysis identified five robust predictors (PLXDC2, DTNB, ALOX5, MALT1, and F13A1), with the SVM model showing optimal performance (AUC = 0.895) and an independently validated nomogram (AUC = 0.933). MR and GEO datasets (GSE138260) further confirmed MALT1 as a potential risk biomarker, and *in vivo* characterization supported its association with adverse prognosis in AD.

**Conclusion:**

This study demonstrates a causal association between CSF metabolites and AD risk, highlighting MALT1 as a promising biomarker and potential therapeutic target for AD.

## Introduction

Alzheimer’s disease (AD) is the leading cause of dementia, characterized by progressive cognitive decline, and imposes a significant socio-economic burden globally (1). More than 57 million people had dementia worldwide, with AD accounting for approximately 60–70% of these cases (2). As the global population ages, the prevalence of AD continues to rise, yet few treatments are available (3). The typical pathological features of AD include the deposition of amyloid β (Aβ) plaques in the brain, which form extracellular deposits, neurofibrillary tangles (NFTs) resulting from the hyperphosphorylation of tau protein within neurons, as well as neuronal apoptosis and synaptic loss caused by increased inflammation and oxidative stress (4). Before the advent of biomarkers, a definitive diagnosis of AD could only be established through post-mortem examination (1). Currently, early diagnosis of AD relies on several approaches, including clinical assessment, imaging, and peripheral biomarkers (5). However, clinical symptoms typically emerge at relatively advanced stages, neuroimaging is costly and subject to individual variability, and peripheral biomarkers often lack sufficient specificity and sensitivity (6). In contrast, cerebrospinal fluid (CSF) biomarkers directly reflect neuropathological alterations, providing high sensitivity and specificity, and thus represent an indispensable tool for early and even preclinical AD diagnosis (7). Identifying CSF biomarkers remains a key focus and hotspot in AD research.

Classical CSF biomarkers, primarily based on Aβ and tau biomarkers, have been confirmed to play a crucial role in AD diagnosis. Compared with cognitively normal elderly individuals, AD patients exhibit a significant reduction in CSF Aβ42 levels (8). Although CSF Aβ40 levels show no obvious change, the CSF Aβ42/Aβ40 ratio is markedly lower in AD patients than in healthy controls (9). Compared with Aβ42 alone, the ratios of Aβ42/Aβ40 and Aβ42/p-tau demonstrate superior performance in both the diagnosis and differential diagnosis of AD (10). Over 70 phosphorylation sites have been identified on tau protein, among which CSF p-tau199 and p-tau217 are markedly increased in AD, serving as indicators of neurodegenerative severity (11). Moreover, recent metabolomic studies have identified significant alterations in CSF metabolites, which are implicated in AD pathogenesis (12). Matrix metalloproteinases (MMPs), such as MMP-9 (13), MMP-2 and MMP-3 (14), which are critical for extracellular matrix turnover, show decreased CSF levels but elevated serum concentrations in AD patients, implying compartment-specific regulation and a possible role as peripheral biomarkers. In the amyloidogenic pathway, β-site amyloid precursor protein cleaving enzyme 1 (BACE1) acts as a key protease responsible for Aβ generation (15). Notably, CSF BACE1 activity is reduced in moderate to severe AD compared with mild cases (16). In addition, such as Kallikrein-8 (17) and Paraoxonase-1 (18), are also significantly altered in both CSF and serum, highlighting their potential as diagnostic indicators. These metabolic changes not only reflect disease reprogramming but also present potential biomarkers and therapeutic targets for AD. However, the study of these CSF biomarkers has been observational, leaving causal relationships inadequately addressed; their diagnostic performance for early AD detection remains to be validated.

Mucosa-associated lymphoid tissue lymphoma translocation protein 1 (MALT1) was initially identified in MALT lymphomas, from which its name is derived (19). The gene is located on human chromosome 18q21, and in certain lymphomas, it can undergo translocation with API2 *t*(11;18)(q21;q21), resulting in constitutive activation of the NF-κB signaling pathway (20). Functionally, MALT1 serves as a paracaspase that acts both as a scaffold protein and a protease within the CARMA1-BCL10-MALT1 (CBM) complex (21). MALT1 plays a critical role in immune cell activation and inflammatory responses. Aberrant MALT1 signaling has been implicated not only in lymphomas but also in autoimmune disorders and chronic inflammatory diseases, highlighting its broad significance in human pathology (22). Despite extensive research in these contexts, the role of MALT1 in neuroinflammation and neurodegenerative diseases, including AD, remains largely unexplored.

In this study, we aim to (i) identify CSF associated with AD; (ii) investigate the role of CSF biomarkers in early AD diagnosis; and (iii) explore novel CSF metabolites as potential diagnostic and therapeutic targets. To achieve these objectives, we employed a multi-omics approach integrating MR, transcriptomic profiling, immune infiltration analysis, and machine learning techniques. Furthermore, MALT1 expression was assessed in the CSF of AD patients, and *in vivo* experiments in mice were performed to investigate the functional impact of MALT1 in AD. Collectively, our findings provide compelling evidence that MALT1 is a key CSF-associated factor that may promote AD pathogenesis.

## Methods

### Study design

**Figure 1:**
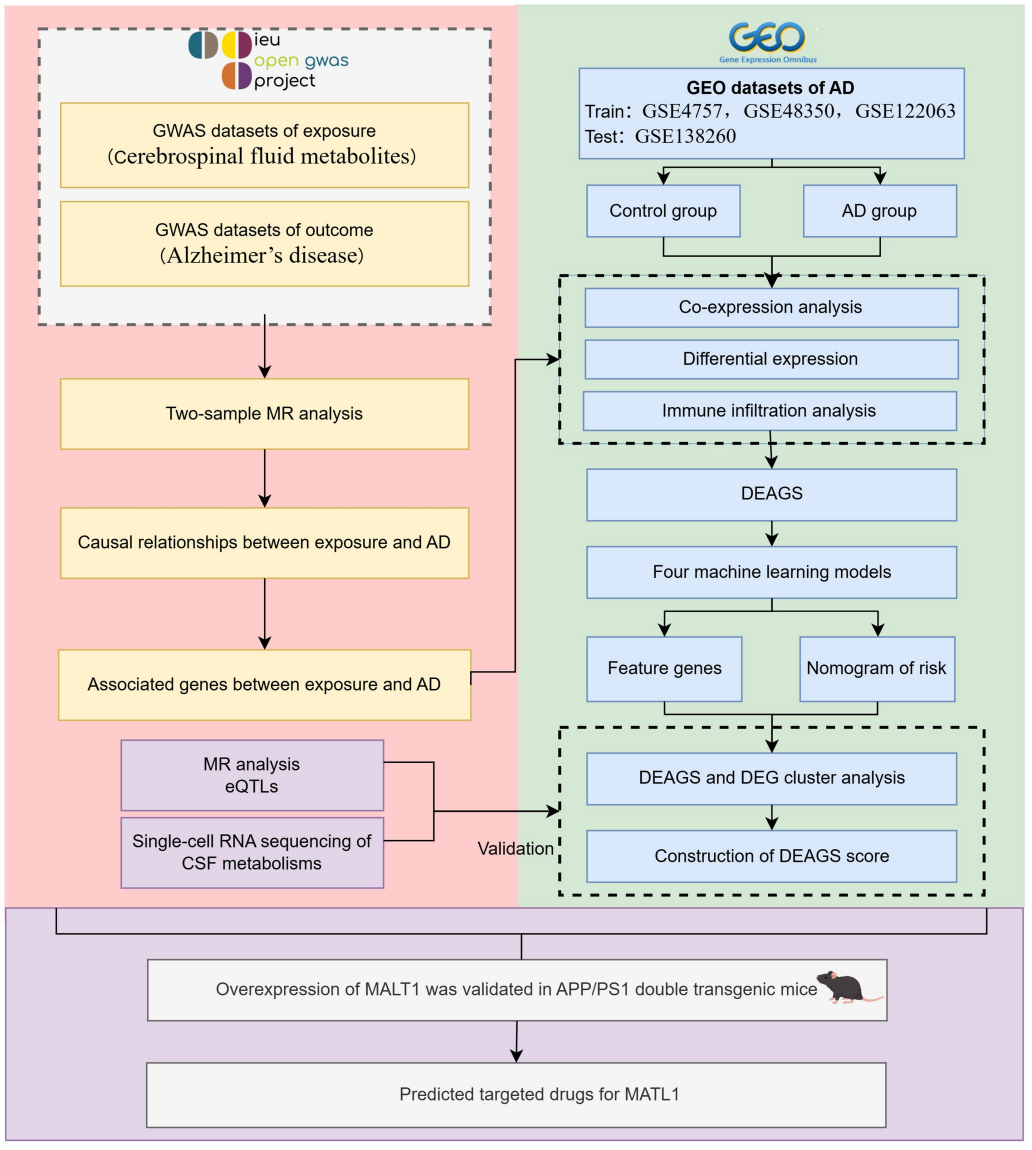
Flowchart of this study design. DEAGS indicates differentially expressed associated gene; DEG, differentially expressed gene; eQTL, expression quantitative trait loci; GEO, Gene Expression Omnibus; GWAS, genome-wide association study; AD, Alzheimer’s disease; and MR, Mendelian randomization.

Two-sample MR analysis was first performed to investigate the causal relationships between differential CSF-related traits and AD. Linkage disequilibrium score regression was then applied to estimate genetic correlations between CSF traits and AD. Gene expression datasets of AD were retrieved from the Gene Expression Omnibus (GEO), including one training set and one validation set with both AD and control samples. Differential expression, correlation, and immune infiltration analyses were performed on candidate genes. Machine learning methods were subsequently used to identify CSF and AD-associated feature genes, and the resulting models were validated using the independent dataset. Unsupervised clustering of AD samples was carried out to further explore the functional mechanisms of exposure-related genes. Finally, *cis*-expression quantitative trait loci (*cis*-eQTLs) corresponding to the identified feature genes were obtained, and MR analyses were performed to evaluate the causal effects of their gene and protein expression levels on AD. To draw valid causal inferences, MR analyses require three key assumptions: the genetic variants must be strongly associated with the exposure, independent of confounding factors, and influence the outcome solely through the exposure. In this study, we adhered to the STROBE-MR reporting guidelines (Strengthening the Reporting of Observational Studies in Epidemiology Using Mendelian randomization, Supplementary material 1), and the overall study design is summarized in Figure 1.

### Mendelian randomization

Genetic instruments for CSF-related traits were selected using SNPs associated at a significance threshold of *p* < 5 × 10^−8^ (due to the limited number of SNPs identified for certain cytokines when they were considered as exposures, we opted for a higher cutoff of *p* < 5 × 10^−6^ instead of *p* < 5 × 10^−8^). Linkage disequilibrium clumping was applied (*R*^2^ < 0.001, window size = 10,000 kb), and ambiguous or palindromic SNPs as well as those with a minor allele frequency < 0.01 were excluded. Potential pleiotropic variants were removed with the MR-PRESSO method, and SNPs associated with confounders were discarded. Data on 338 CSF metabolites were sourced from a metabolome-wide association study by Daniel J. Panyard et al., involving 291 individuals of European descent (23). Outcome data for AD were obtained from publicly available Finngen datasets, including 6,741 cases and 428,053 controls. The Cochran Q-test for heterogeneity, utilized in the IVW method, revealed no significant heterogeneity in our study (*p* > 0.05). The non-significant result of the Cochran Q-test enhances the study’s validity by showcasing the homogeneity of the genetic instruments employed (Guidelines for performing Mendelian randomization investigations: update for summer 2023).

### Mapping SNPs to genes and analyses of differentially expressed associated genes

The web-based variant annotation tool SNP^1^ was used to associate each queried SNP with its closest gene, which could be overlapping, upstream, or downstream (NPnexus: a web server for functional annotation of human genome sequence variation). Three GEO datasets (GSE4757, GSE48350, and GSE122063) were analyzed, including both control and experimental samples, while GSE138260 was employed for validation. Genes derived from SNP mapping were intersected with those from the GEO datasets to identify differentially expressed genes (DEGs), which were subsequently subjected to further analyses to obtain DEAGs. The expression levels of genes in the control and AD groups were extracted from the merged GEO dataset for differential expression analysis. Genes with a *p*-value < 0.05 were defined as differentially expressed associated genes (DEAGs). The expression results were visualized using box plots. Chromosomal localization of DEAGs was performed with Perl scripts, and their genomic positions were displayed as a heatmap. Furthermore, pairwise correlations between DEAGs were calculated using the cor function, and the correlation results were visualized accordingly.

### Analysis of immune cells in AD samples

To investigate immune cell infiltration, the relative proportions of RNA transcript subsets were estimated using the cell type identification tool (12). A total of 1,000 simulations were conducted to obtain the relative abundance of immune cell populations, normalized to a total of one, and the results were displayed as bar plots. Subsequently, single-sample gene set enrichment analysis (ssGSEA) was conducted to compare the immune cell compositions between the control and AD groups, and the results were displayed as box plots. Correlation analysis was then performed between DEAGs and ssGSEA scores, yielding correlation coefficients, which were visualized as a heatmap to illustrate the relationships between immune cells, genes, and immune cell–gene interactions.

### Single-cell RNA sequencing data analysis

We analyzed single-cell RNA sequencing data from CSF samples in the GEO dataset GSE200164, which includes 59 participants—45 healthy controls and 14 individuals with mild cognitive impairment or AD. Downstream analyses were performed in R using the Seurat package (v4.0). After rigorous quality control, high quality cells were retained for subsequent analyses. Data normalization was conducted using the SCTransform method, and batch effects were corrected with the Harmony algorithm. Based on principal component analysis and the Louvain clustering algorithm, we identified 13 distinct cellular populations at a resolution of 0.1. Using canonical marker gene expression profiles, these clusters were annotated as naïve CD4^+^ T cells, CD8^+^ effector T cells, CD4^+^ T cells, regulatory T cells, innate-like T/MAIT cells, natural killer cells, innate lymphoid cells, B cells, dendritic cells, plasmacytoid dendritic cells, monocytes, microglia, and proliferating cells. We further compared the proportions of each cell type between healthy controls and disease groups, and assessed the distribution of differentially expressed genes across immune cell subsets.

### Identification of the feature genes and construction and validation of a nomogram based on ML

Based on the expression profiles of differentially expressed associated genes (DEAGs), four machine learning algorithms—Random Forest (RF), Support Vector Machine (SVM), Generalized Linear Model (GLM), and Extreme Gradient Boosting (XGBoost)—were applied to construct predictive models. Model performance was evaluated using residual boxplots and receiver operating characteristic (ROC) curves, and feature genes were identified according to the optimal model. A nomogram was then developed based on the expression levels of the selected feature genes in the AD and control groups. Calibration curves and decision curve analysis (DCA) were used to assess predictive accuracy and clinical utility, while ROC analysis in an independent dataset was performed for external validation (24). To explore potential causal relationships, cis-expression quantitative trait loci (cis-eQTLs) of the feature genes were subjected to MR analysis using standard methods, including inverse variance weighted (IVW) and MR-Egger regression.

### Animals and experimental design

APP/PS1 double transgenic mice (Nanjing Zhishu Yantu Biotechnology, China) were used as a well-established model of AD. These mice co-express human amyloid precursor protein (APP) and presenilin-1 (PS1) genes harboring familial AD mutations, leading to accelerated Aβ deposition and the development of hallmark AD pathology. C57BL/6J wild-type mice served as controls to exclude confounding effects of genetic background or age. Neuronal overexpression of MALT1 was induced by stereotaxic delivery of an adeno-associated viral (AAV) vector into the cortical region, ensuring region- and cell-type-specific expression. All animals were maintained under specific pathogen-free (SPF) conditions in a temperature- and humidity-controlled environment with a 12-h light/dark cycle and ad libitum access to food and water. All animal experiments were conducted in accordance with institutional ethical guidelines and approved by the Animal Care and Use Committee of Xiangya Third Hospital, Central South University (No. APU-2025-0389).

### Western blot analysis

The CSF proteins were extracted, quantified by the BCA assay, and equal amounts were separated by SDS-PAGE. Following transfer to a PVDF membrane, the membrane was blocked and incubated overnight at 4 °C with a primary antibody against MALT1 (Invitrogen, Catalog No. PA5-114500, 1:500). After washing, the membrane was incubated with a horseradish peroxidase-conjugated secondary antibody (1:5000) for 1 h. Protein bands were detected using chemiluminescence and visualized with a Bio-Rad ChemiDoc system. Band intensities were quantified using ImageJ software and normalized to GAPDH as a loading control.

### Hematoxylin and eosin (H&E) staining

H&E staining was employed to examine the histological characteristics of brain tissues in a middle cerebral artery occlusion and reperfusion (MCAO/R) mouse model. Both normal and injured brain tissues were fixed in 10% formalin, embedded in paraffin blocks, and sectioned into 5-micron-thick slices, which were then placed on glass microscope slides. Following deparaffinization and rehydration, the sections were stained with hematoxylin to highlight cell nuclei, and then counterstained with eosin to enhance cytoplasmic visualization. The stained sections were subsequently examined under a microscope, and images were captured for further analysis.

### Nissl staining

To prepare for Nissl staining, slides containing adherent tissue sections were placed in a constant temperature drying oven at 60 °C for 30 min to 1 h, ensuring that the tissues adhered securely to the glass slides. Dewaxing was performed using two rounds of xylene (15 min each), followed by gradient dehydration in ethanol: 100% (twice), 95, 90, 80, 70, and 50%, with each step lasting 5 min. Slides were rinsed three times with distilled water, each rinse lasting 5 min. The slides were then placed in a 60 °C incubator and stained with 1% toluidine blue for 40 min (or with tar purple for 30 s). After staining, excess dye was gently rinsed off with distilled water (approximately 10 s). Slides were then washed in distilled water several times (at least three times) to completely remove the acidic differentiating solution and halt the differentiation process, with each wash lasting about 10–20 s. The slides were then sequentially dehydrated in 70, 80, 95, and 100% ethanol, followed by clearing in xylene. Finally, slides were mounted with neutral resin for microscopic examination.

### Immunofluorescence staining

Brain tissues from the mouse model were harvested and fixed in 4% formaldehyde for 15 min, followed by three washes with phosphate-buffered saline (PBS). The tissues were then permeabilized at room temperature with 0.5% Triton X-100 for 20 min to enhance antibody penetration. After permeabilization, a specific antibody targeting MALT1 was applied for overnight incubation at 4 °C. The next day, DAPI was used to stain the cell nuclei in the dark for 30 min. Following this, fluorescently labeled secondary antibodies were added to the tissue sections. Finally, fluorescence was observed using a fluorescence microscope to assess the localization and expression levels of MALT1 in the brain tissues.

### Mitochondrial immunofluorescence staining

Dilute the MitoTracker probe to the working concentration in DMEM culture medium or PBS buffer (e.g., MitoTracker Green at 20–200 nM, MitoTracker Red and MitoTracker Deep Red at 25–500 nM). Discard the culture medium and add the pre-warmed MitoTracker staining solution, incubating at 37 °C for 15–45 min. Then, add pre-warmed 4% paraformaldehyde to fix the tissues for 10–15 min. Discard the fixative and wash the tissues with PBS buffer 2–3 times. Next, add 0.3% Triton X-100 to permeabilize the tissues for 10 min, followed by washing the tissues again with PBS buffer 2–3 times. Add DAPI or Hoechst dye to stain the nuclei. After staining, wash the tissues 2–3 times with PBS buffer and then observe the tissues using a fluorescence microscope, confocal microscope, or super-resolution microscope to record the mitochondrial fluorescence signals. Analyze the parameters such as mitochondrial morphology, distribution, and fluorescence intensity.

### Molecular docking

In an effort to improve the prognosis and overall survival rate of the disease, we attempted to investigate the interaction between drugs and MALT1. The molecular docking program AutoDock Tools (25) and AutoDock vina (26) were used for the automated molecular docking simulations. The 3D structure of MALT1 (3 V55) protein was obtained from Uniprot database^2^. We have used Discovery Studio Visualizer to visualize and analyze the interactions (27). Prior to molecular docking calculations, water molecules and the ligand in the protein structure were removed, and hydrogens and Gasteiger charges were added. Eight docking poses were obtained for molecular docking calculation.

### Image analysis

All microscopic images and Western blot bands were quantified using ImageJ software. Data are presented as the mean ± standard deviation (SD). Statistical comparisons between two groups were performed using Student’s *t*-test, while multiple-group comparisons were analyzed by one-way analysis of variance (ANOVA). *p*-value < 0.05 was considered statistically significant.

### Statistical analysis

This study was mainly done using R V4.2.1. In the MR analysis, to correct for multiple testing, we applied the Bonferroni correction to adjust the significance level thresholds for multiple analyses. In the bioinformatics part, we used Strawberry Perl 5.32.1.1 to merge GEO data sets and extract and annotate data. For 2 independent samples, we applied a t test, while for 2 paired samples, we used the Wilcoxon paired rank-sum test, and for ≥3 groups of data, we used ANOVA and the Kruskal-Wallis rank-sum test. The Spearman rank correlation test was used for correlation analysis. We set a *p* value < 3.3 × 10^−3^ (0.05/15) after correction by the Bonferroni correction method as being statistically significant.

## Results

### MR analysis between cerebrospinal fluid metabolites and Alzheimer’s disease

**Figure 2:**
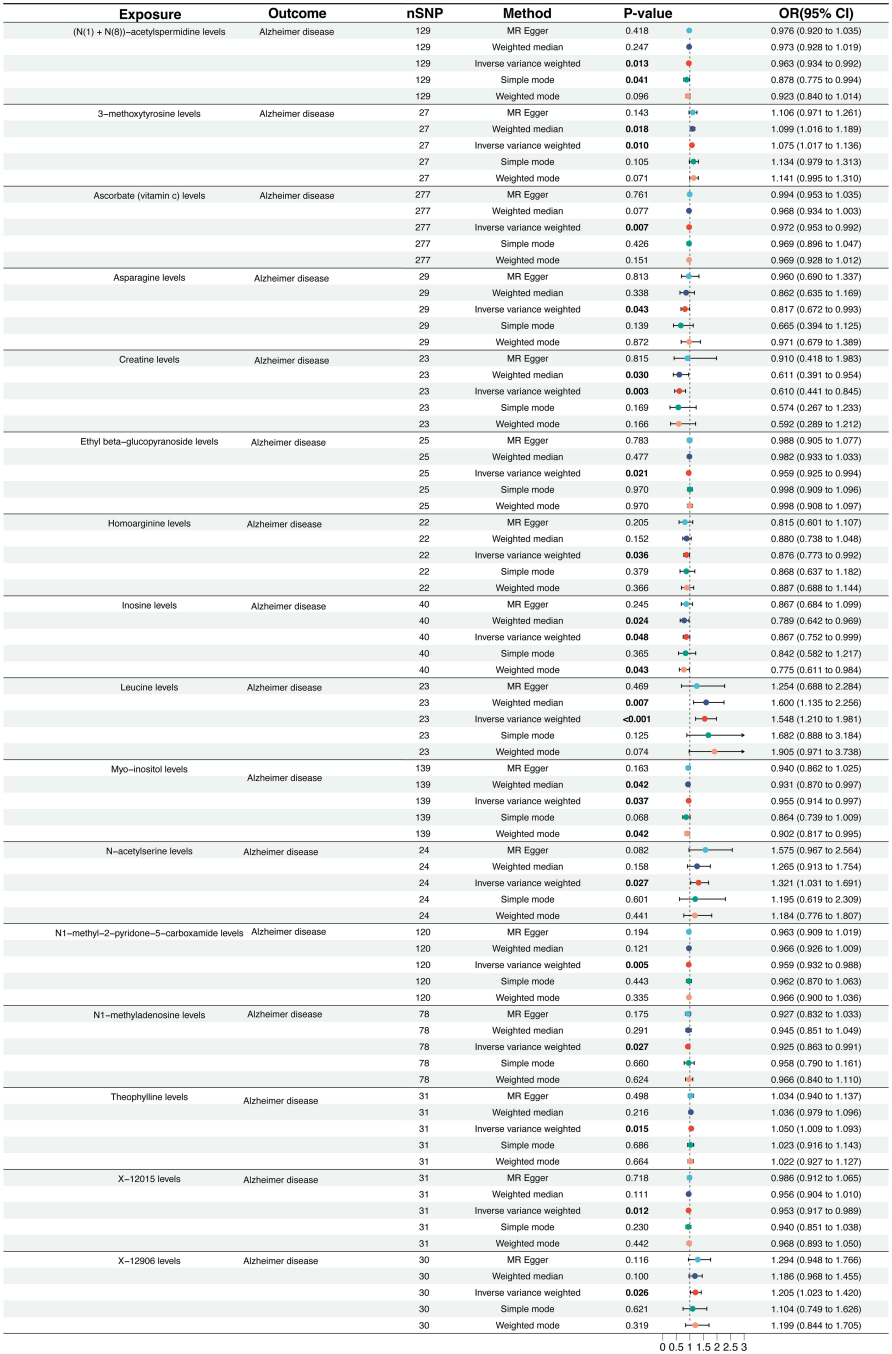
Forward Mendelian randomization estimates of circulating metabolites on Alzheimer’s disease risk. Forest plot showing the causal effects of genetically predicted circulating metabolites on the risk of Alzheimer’s disease. Odds ratios (ORs) with 95% confidence intervals (CIs) were estimated using multiple MR approaches, including inverse variance weighted (IVW), MR-Egger, weighted median, simple mode, and weighted mode. Each row represents one metabolite, and the square size indicates the precision of the estimate. The vertical line at OR = 1 represents the null hypothesis of no causal effect.

**Figure 3:**
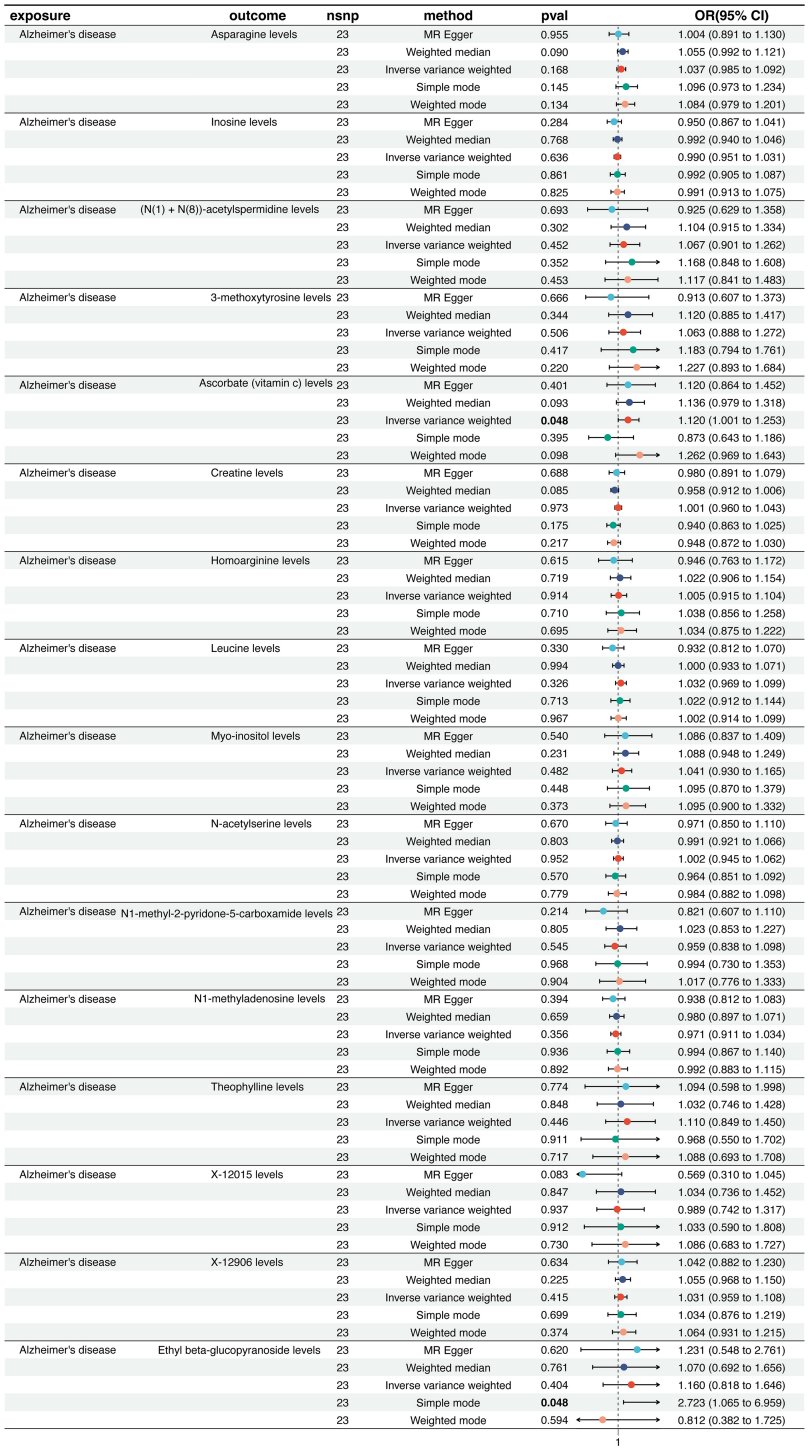
Reverse Mendelian randomization estimates of Alzheimer’s disease liability on circulating metabolite levels. Forest plot showing the causal effects of genetic liability to AD on circulating metabolites. ORs with 95% CIs were estimated using the same MR approaches as in Figure 2.

We conducted two-sample MR to assess potential causal associations between differential CSF metabolites and AD risk in the forest plot (IVW as primary, complemented by weighted median, MR-Egger, and mode-based estimators) (Supplementary Table S1). The forward MR analysis identified protective effects of creatine, homoarginine, and *N*-acetylserine, while leucine, inosine, N1-methyladenosine, and 3-methoxytyrosine increased AD risk (Figure 2). Specifically, elevated genetically predicted levels of leucine (OR = 1.548, 95% CI: 1.210–1.981, *P* < 0.001) were significantly associated with an increased risk of AD, implicating pathways related to purine metabolism, RNA modification, and catecholamine metabolism. In contrast, higher levels of creatine (OR = 0.610, 95% CI: 0.441–0.845, *p* = 0.003) were protective against AD, suggesting beneficial effects of energy buffering and amino acid metabolism. Other metabolites, such as ascorbate, leucine, and myo-inositol, showed nominal associations but did not reach statistical significance after multiple-testing correction (Supplementary Tables S2, S3). The funnel plot visually evaluates the absence of potential bias arising from genetic variant heterogeneity, specifically horizontal pleiotropy (Supplementary Figure S1). Finally, a reverse MR analysis was conducted to evaluate the plausibility of reverse causation between AD and the CSF identified in our study (Figure 3). In the reverse MR analysis, AD liability did not demonstrate consistent causal effects on most circulating metabolites. Collectively, these results provide evidence that specific metabolic alterations exert a causal influence on AD susceptibility. In particular, perturbations in purine and amino acid metabolism emerge as critical metabolic axes in AD pathogenesis, highlighting their potential as early biomarkers and therapeutic targets.

### Bulk RNA data analysis

**Figure 4:**
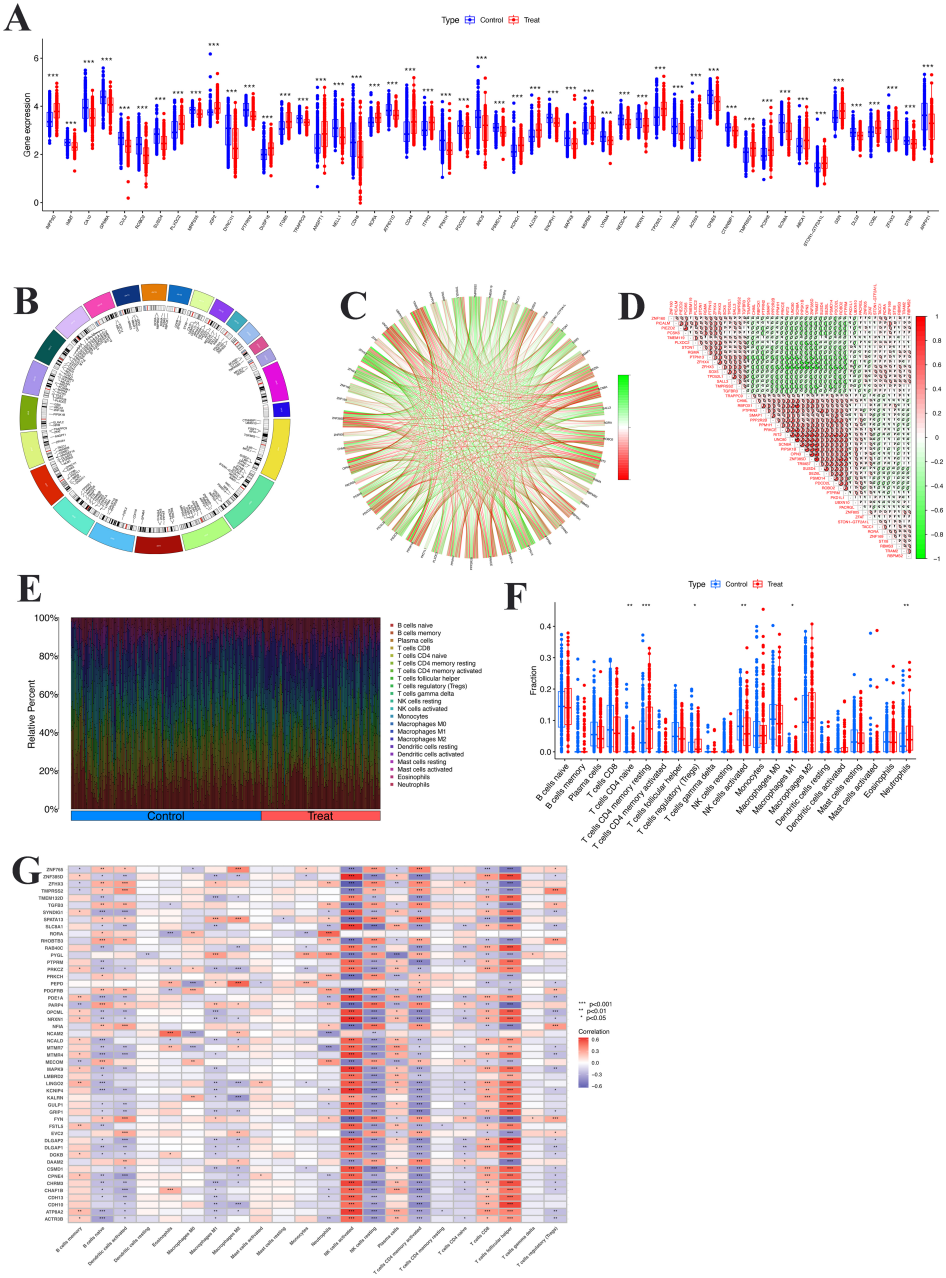
Integrated analysis of immune-related gene expression and immune cell infiltration. **(A)** Differential expression of immune-related genes between control (blue) and treatment (red) groups. **(B)** Chromosomal distribution of immune-related genes. **(C)** Gene–gene interaction network, with green and red lines indicating positive and negative correlations, respectively. **(D)** Correlation matrix of immune-related gene expression. **(E)** Relative fractions of 22 immune cell types in control and treatment groups estimated by CIBERSORT. **(F)** Comparison of immune cell abundances between groups. **(G)** Heatmap showing correlations between representative immune-related genes and infiltrating immune cells. *P < 0.05, **P < 0.01, ***P < 0.001.

In differential expression analysis, we identified the top 50 differentially expressed genes (DEAGs) significantly associated with AD as shown in Figure 4A. The results showed significant differences in the expression levels of these genes between the experimental and control groups, with some genes significantly upregulated (e.g., INPP5D, TCAP, CUL2, ROBO2, and PLXDC2) and others significantly downregulated (e.g., PMPCA, ANGPT1, NELL1, CDH8, and ROR1). These genes play important roles in multiple biological pathways, including lipid metabolism, inflammatory response, cell adhesion, and neural signaling. These genes were widely distributed across multiple chromosomes, with notable clusters on chromosomes 1, 7, 11, and 17 (Figure 4B). Notably, these clusters contain genes implicated in amyloid precursor protein processing (APP, PSEN2), tau pathology, lipid metabolism (APOE), and neuroinflammation, highlighting chromosomal hotspots that converge on core pathogenic pathways of AD. To further explore the relationships among these genes, correlation analysis was performed (Figures 4C,D). Several genes, including PICALM, TMEM119, PIEZO2, and PLXDC2, were consistently upregulated in disease samples, while members of the ZNF gene family (e.g., ZNF385D, ZNF160, ZNF169, and ZNF805) and synaptic or immune-related genes (e.g., RGMA and PTPRN2) were downregulated.

### Immune cell infiltration and treatment-induced remodeling

To investigate the immune microenvironment in AD, we performed immune cell deconvolution to assess the relative fractions of 22 immune cell subsets. As shown in Figure 4E, the overall immune landscape differed markedly between the Control and AD groups. AD samples displayed a distinct redistribution of both adaptive and innate immune populations, reflecting broad immune dysregulation. Detailed comparison of cell fractions revealed significant alterations in multiple immune subsets (Figure 4F). In adaptive immunity, the AD group exhibited significantly increased proportions of regulatory T cells (Tregs), activated CD4^+^ memory T cells, and plasma cells, while naïve B cells and resting CD4^+^ memory T cells were reduced. These findings suggest abnormal activation of adaptive immune responses accompanied by altered regulatory control in AD. In the innate compartment, AD samples showed a reduced proportion of M0 macrophages with polarization toward both M1 (pro-inflammatory) and M2 (anti-inflammatory) phenotypes, indicating immune activation and functional remodeling. In addition, the frequencies of activated NK cells and activated dendritic cells were significantly elevated, consistent with a more activated innate immune state. To explore the molecular basis of these immune alterations, we analyzed correlations between differentially expressed genes and immune cell subsets (Figure 4G). AD-associated risk genes, including APP, PICALM, and RBFOX1, were positively correlated with plasma cells, activated CD4^+^ T cells, and Tregs, linking amyloid-related pathways to adaptive immune activation. In contrast, members of the ZNF family (e.g., ZNF385D, ZNF423, and ZNF765) showed negative correlations with naïve B cells and resting CD4^+^ memory T cells, suggesting divergent transcriptional regulation of immune homeostasis. Furthermore, genes such as RORA, RGMA, and TMPRSS2 were positively correlated with activated macrophages and dendritic cells, supporting their involvement in microglial activation and neuroinflammation.

### Single-cell RNA sequencing reveals cell-specific expression of AD-associated genes in cerebrospinal fluid

**Figure 5:**
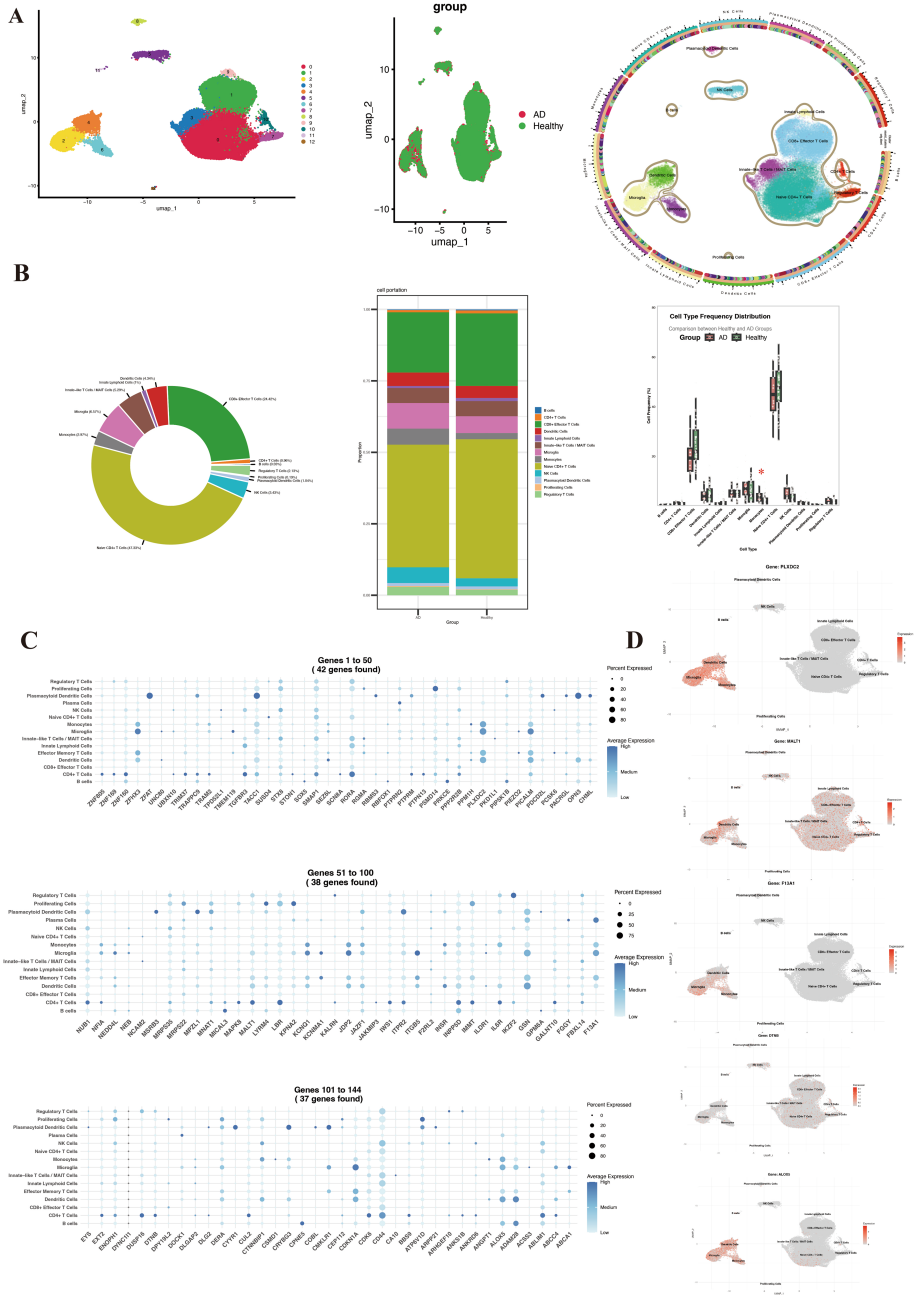
Single-cell immune profiling of cerebrospinal fluid reveals Alzheimer’s disease-associated cellular composition and gene expression signatures. **(A)** UMAP visualization colored by patient group (healthy controls vs. AD), sample origin, and annotated cell types. **(B)** Comparative distribution of immune cell subsets between the two groups. **(C)** Specific expression patterns of differentially expressed genes across immune cell subsets. **(D)** Spatial expression localization of key genes (ALOX5, PLXDC2, F13A1, DTNB, and MALT1) in UMAP projection.

Analysis of CSF single-cell RNA sequencing data from the GSE200164 dataset (45 healthy controls, 14 AD patients) identified the distribution of differentially expressed genes across CSF immune cell populations. UMAP visualization delineated 13 distinct immune cell populations based on transcriptomic signatures (Figure 5A), including major subsets such as naive CD4 + T cells (32,136 cells), CD8 + effector T cells (16,577 cells), regulatory T cells (1,445 cells), B cells (227 cells), dendritic cells (2,948 cells), monocytes (2,014 cells), and microglia (4,458 cells). Cell proportion analysis indicated similar immune cell composition in AD patients and healthy controls, with the exception of monocytes (Figure 5B). Differentially expressed genes exhibited distinct distribution patterns across immune cell subsets (Figure 5C), suggesting potential roles in regulating specific immune functions, which validates the correlation between these differentially expressed genes and immune cells. Importantly, the study precisely localized five AD-associated genes to specific cell types (Figure 5D). ALOX5, PLXDC2, and F13A1 were primarily enriched in myeloid cell populations (dendritic cells, microglia, and monocytes), suggesting their involvement in AD pathogenesis through modulation of innate immunity. In contrast, DTNB and MALT1 did not demonstrate strong cell type-specific expression, suggesting broader cellular functions. These findings provide key cellular-level insights into the role of AD-associated genes within the CSF immune microenvironment.

### Machine learning model performance and construction of a predictive nomogram

**Figure 6:**
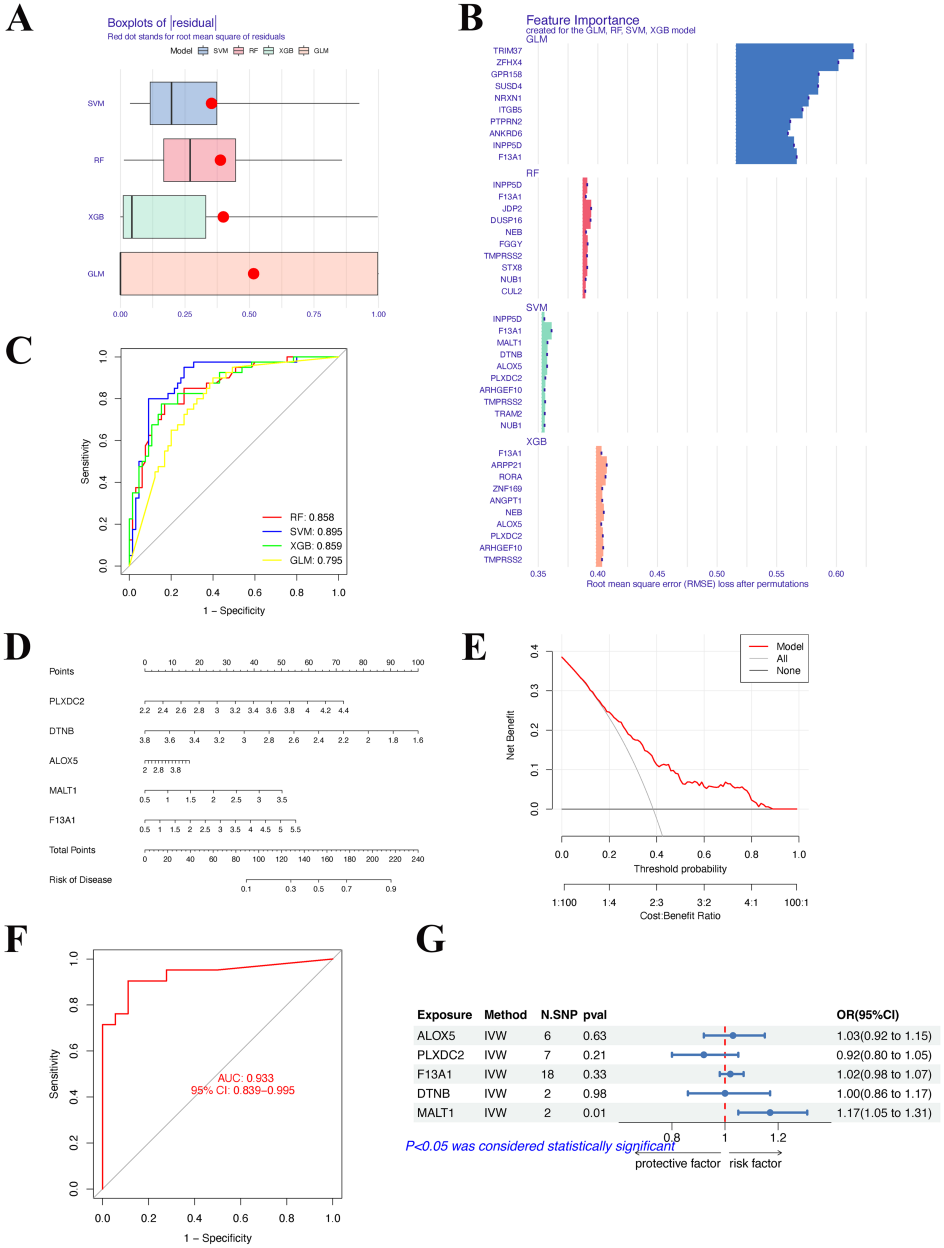
Machine learning–based identification and validation of hub genes. **(A–C)** Performance of four machine learning models (SVM, RF, XGB, and GLM) in feature selection and disease prediction. **(D)** Nomogram integrating five hub genes (PLXDC2, DTNB, ALOX5, MALT1, and F13A1) for individualized risk assessment. **(E,F)** Clinical utility and predictive accuracy of the nomogram assessed by decision curve analysis and ROC curve. **(G)** Mendelian randomization indicated MALT1 was a potential genetic risk factor.

We next applied four machine learning algorithms—support vector machine (SVM), random forest (RF), extreme gradient boosting (XGB), and generalized linear model (GLM)—to evaluate the predictive potential of differentially expressed genes in AD. Residual analysis demonstrated that SVM and XGB achieved the lowest errors, indicating superior stability compared with RF and GLM (Figure 6A). Feature importance analysis identified several robust predictors across models, including ALOX5, PLXDC2, F13A1, DTNB, and MALT1 (Figure 6B). Receiver operating characteristic (ROC) analysis further confirmed that SVM provided the highest discriminative performance (AUC = 0.895), followed by XGB (0.859), RF (0.858), and GLM (0.795) (Figure 6C). Based on the top five predictive genes, we constructed a nomogram for individualized risk estimation (Figure 6D). The nomogram demonstrated good calibration and provided a clinically interpretable tool for patient-level risk stratification. Decision curve analysis showed that the nomogram yielded a higher net clinical benefit across a wide range of threshold probabilities compared with “treat all” or “treat none” strategies (Figure 6E). Importantly, the predictive performance of the model was validated in an independent dataset, achieving an AUC of 0.933 (95% CI: 0.839–0.995), underscoring its robustness and translational potential (Figure 6F).

### MR analysis using predicted genes identified genes for AD

We used MR to explore the potential genetic associations of ALOX5, PLXDC2, F13A1, DTNB, and MALT1 with AD in Figure 6G. MR revealed a statistically significant causal association between MALT1 and AD (OR = 1.17, 95% CI = 1.05–1.31, *p* = 0.01) and MVMR to correct for potential effects of AD risk factors. Although the role of F13A1 was analyzed, no significant association was found between F13A1 and AD. This indicates that the causal effect estimate of F13A1 may be close to zero, or its error margin is too large to conclude statistical significance. ALOX5, PLXDC2, and DTNB were excluded due to an insufficient number of available SNPs (after standardization) and lacked available eQTL data. This result suggests that MALT1, as a crucial CSF metabolite, promoted AD progression by enhancing exposure effects (e.g., inflammation/matrix degradation), providing evidence for functional validation and target research.

### MALT1 overexpression exacerbates neuroinflammation, neuronal injury, and mitochondrial dysfunction in the AD mouse model

**Figure 7:**
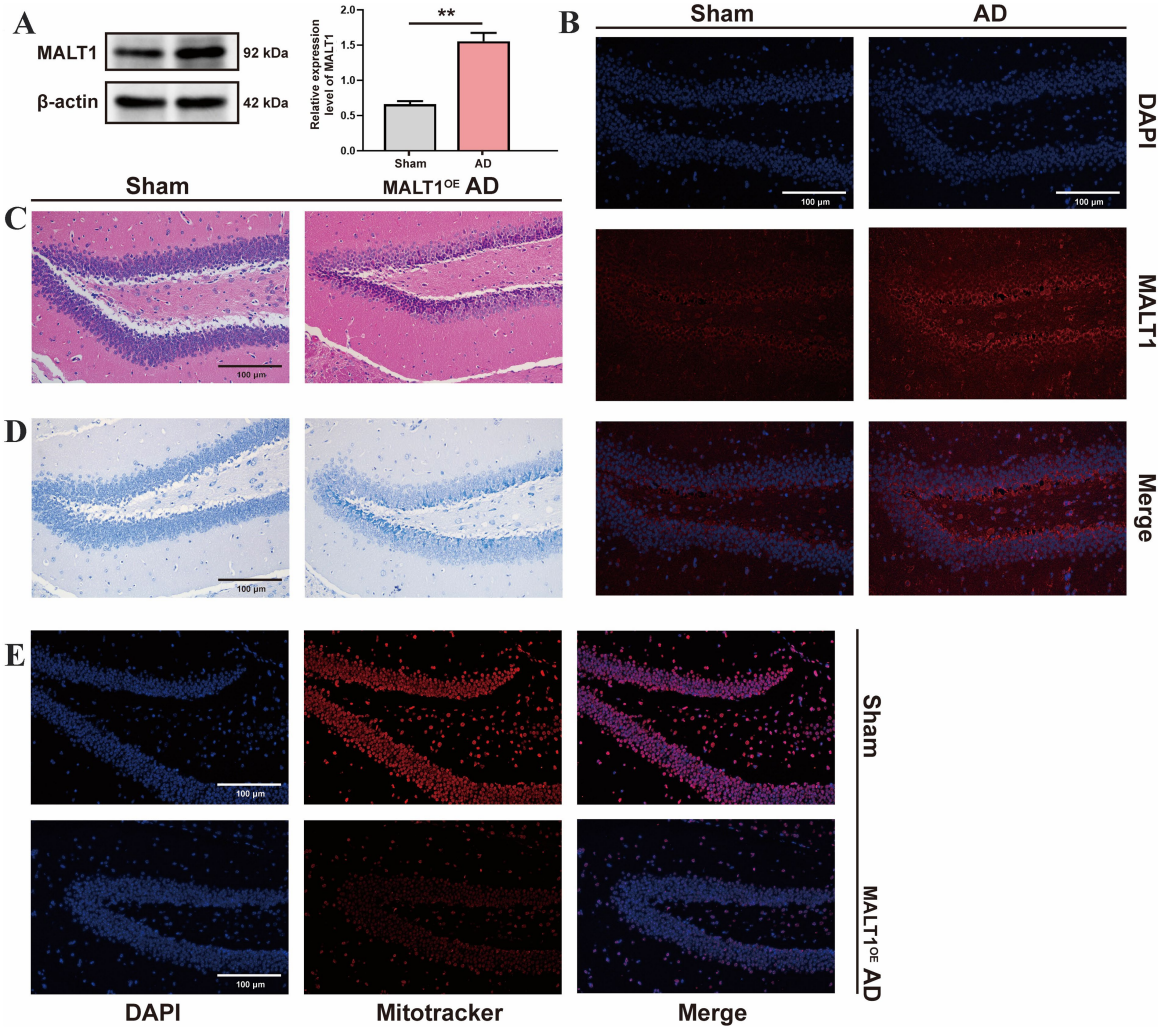
MALT1 overexpression induces inflammatory and neuronal damage in the AD mouse model. **(A)** Western blot analysis of MALT1 expression (Invitrogen, PA5-114500, 1:500, molecular weight 92 kDa) in CSF from AD model mice and control, showing higher MALT1 expression in the AD group. **(B)** Immunofluorescence staining of MALT1 expression, with increased MALT1 levels observed in the AD group. **(C)** H&E staining showing the extent of inflammatory infiltration in brain tissue; more pronounced inflammation in the AD overexpression MALT1 group. **(D)** Nissl staining to assess neuronal damage, revealing more severe neuronal injury in the AD overexpression MALT1 group. **(E)** Mitochondrial immunofluorescence staining demonstrates worse mitochondrial condition in the AD overexpression MALT1 group. **P < 0.01.

To further clarify the changes in MALT1 expression within CSF and brain tissue of AD, we established an AD mouse model. Compared with the control group, Western blot analysis demonstrated a significant elevation of MALT1 protein expression in the CSF of the AD group (*p* < 0.05, Figure 7A). Consistently, immunofluorescence staining further confirmed this finding, showing markedly enhanced MALT1 fluorescence intensity predominantly localized in the cytoplasm of neurons in the AD group (Figure 7B). These results demonstrate that MALT1 expression is markedly upregulated in the AD model. Moreover, H&E staining showed extensive inflammatory cell infiltration and tissue damage in the brains of MALT1 overexpressing AD mice, characterized by pronounced perivascular inflammation and glial activation, whereas the control group exhibited only mild inflammatory changes (Figure 7C). Nissl staining revealed disorganized neuronal architecture and a marked reduction or loss of Nissl bodies in the AD group, indicative of neuronal degeneration, while neurons in the control group remained morphologically intact with abundant Nissl substance (Figure 7D). Furthermore, mitochondrial immunofluorescence demonstrated that mitochondria in the AD group were fragmented and swollen with reduced fluorescence intensity, suggesting impaired mitochondrial integrity and function, whereas the control group displayed a well-preserved, reticular mitochondrial network (Figure 7E). Collectively, these findings indicate that MALT1 overexpression aggravates neuroinflammation, neuronal injury, and mitochondrial dysfunction in the AD mouse model.

### Identification of potential drugs targeting the MALT1 gene via molecular docking analysis

**Figure 8:**
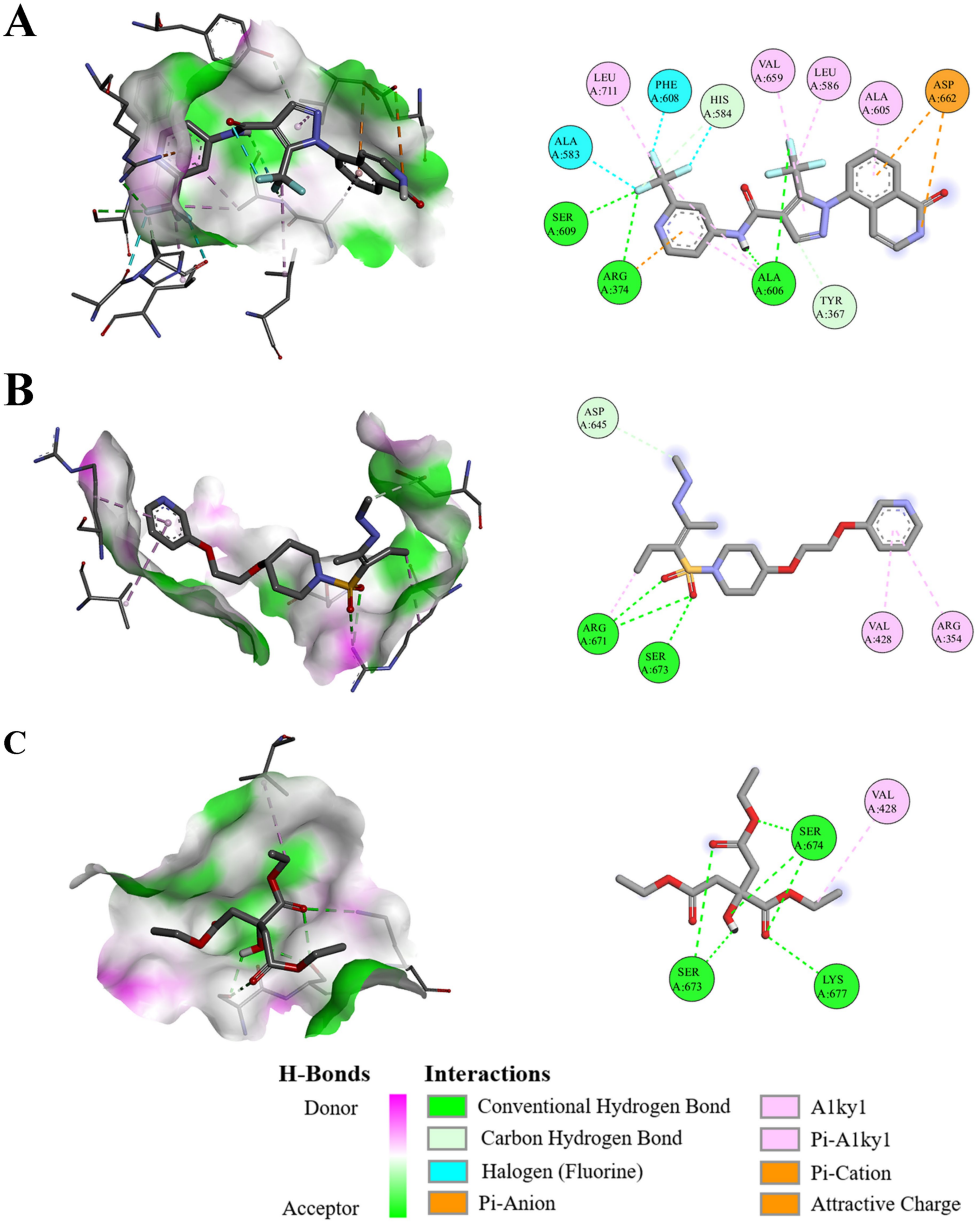
Drugs for the MALT1 gene from GeneCards. After docking each protein with the small molecule, including **(A)** SAFIMALTIB, **(B)** MEPAZINE ACETATE, and **(C)** SGR-1505, the results were visualized with 3D and 2D diagrams. The 3D diagram shows the docking binding pocket, while the 2D diagram represents the interaction forces, with different colors indicating different types of interactions. The letters represent amino acid abbreviations, and the numbers correspond to the amino acid sequences. Binding energies below −5 Kcal/mol are considered usable, with more negative values indicating stronger and more stable protein-ligand binding.

We performed molecular docking of 8 drugs with MALT1 to predict potential MALT1-targeting drugs, including MEPAZINE ACETATE, SAFIMALTIB, PECAZINE, SAFIMALTIB, SGR-1505, Cysteine Guanylate, and Paraffin. The results revealed that Safimaltib (Figure 8A), Mepazine acetate (Figure 8B), and SGR-1505 (Figure 8C) can stably bind to MALT1, respectively with binding energies of −9.0 Kcal/mol, −7.4 Kcal/mol, and −6.1 Kcal/mol. Notably, Safimaltib exhibited the most stable binding to MALT1, interacting with key amino acid residues (Ser609, Arg374, Ala606, and Tyr367) via hydrogen bonds. The molecular docking results suggest that Safimaltib is a potential targeting drug for MALT1, providing a new therapeutic strategy for AD.

## Discussion

This study aims to identify CSF biomarkers for the early detection of AD and to explore potential targets for therapeutic intervention. We employed a comprehensive approach combining MR, transcriptomic profiling, immune cell deconvolution, and machine learning techniques to comprehensively explore the role of CSF metabolites in AD. Our findings provide several important contributions: (i) We provide new insights into the causal role of CSF metabolites in AD pathogenesis; (ii) we identify transcriptomic and immune signatures associated with disease progression and develop a predictive model with potential clinical applicability; (iii) we demonstrate that MALT1 overexpression exacerbates neuroinflammation, neuronal injury, and mitochondrial dysfunction in an AD mouse model, and explore its potential as a therapeutic target through molecular docking analyses.

We first performed MR analysis to identify several CSF metabolites that influence AD risk. The results showed that creatine levels were significantly protective against AD development. Creatine, a key component of the ATP buffering system in the brain, is crucial for maintaining neuronal energy reserves (28). Reduced creatine levels increase neuronal vulnerability, while intraventricular creatine treatment mitigates AD pathology and memory deficits by inhibiting STAT1 phosphorylation (29). Conversely, elevated leucine levels were associated with an increased AD risk. Leucine is a branched-chain amino acid (30). Leucine-rich repeat kinase 2 (LRRK2) mediated phosphorylation of Rab12 and Rab10 contributes to endolysosomal dysfunction in AD, with pS106-Rab12 accumulation in GVBs and co-pathology with tau, suggesting a key role for LRRK2 in tau-related neurodegeneration (31). These findings indicate that CSF dysregulation may favor anabolic processes driven by amino acids, while compromising energy buffering, potentially contributing to neuronal vulnerability and accelerating AD progression. The key metabolites, such as creatine and leucine, provide critical insights into disease pathophysiology and represent promising biomarkers for early diagnosis and potential therapeutic targets, highlighting the central role of CSF in AD detection and intervention strategies.

To investigate the critical role of CSF in AD, we performed integrated transcriptomic and immune analyses. Transcriptomic profiling revealed dysregulated genes mediating metabolic disturbances in AD. Upregulated genes such as INPP5D (32), PICALM (33), TMEM119 (34), and PLXDC2 (35) were linked to amyloid processing, microglial activation, and synaptic function, while downregulated ZNF family members (36), RGMA (37), and PTPRN2 (38) indicated impaired transcriptional regulation and synaptic signaling. These alterations converge on key AD-related pathways, including amyloid metabolism, tau pathology, lipid homeostasis, and neuroinflammation. Immune deconvolution further revealed activation of adaptive and innate immunity, characterized by increased regulatory and memory T cells (39), plasma cells (40), and microglia polarization (41), reflecting chronic neuroinflammation. Correlation analyses connected APP (42), PICALM (43), and RBFOX1 (44) expression with immune activation, suggesting crosstalk between amyloid pathology and immune remodeling. Collectively, these findings indicate that CSF metabolic and transcriptional dysregulation contribute to immune imbalance and AD progression.

Next, MALT1 overexpression was found to induce neuroinflammation, neuronal injury, and mitochondrial dysfunction, highlighting MALT1 as a potential contributor to AD. MALT1 is a critical regulator of various pathophysiological processes, primarily through its modulation of the NF-κB signaling pathway (45). In immune cells, it plays a key role in activating NF-κB in response to antigen stimulation, while in neuronal cells, it is involved in neuroinflammation, especially in diseases like experimental autoimmune encephalomyelitis (EAE), where it contributes to pathogenic Th17 cell function via non-degradative polyubiquitination (46). MALT1 also exacerbates ischemic brain injury and spinal cord ischemia/reperfusion (SCI/R) injury by promoting glial endoplasmic reticulum stress, neuroinflammation, and neuronal damage, with its inhibition alleviating these effects (47). In ischemic stroke, MALT1 regulates GluN2B phosphorylation and calcium overload through its interaction with HECTD4, with downregulation reducing neuronal damage (48). Furthermore, in glioblastoma, MALT1 regulates the mesenchymal phenotype by modulating NF-κB signaling, and its repression by miR-181d shifts the tumor toward a less malignant proneural subtype (49). MALT involvement in inflammation, ischemic injury, and tumor progression underscores its potential as a therapeutic target across a range of diseases. However, research on MALT1 in CSF and brain tissue remains limited in AD, and its precise role in neuroinflammation and neurodegenerative diseases is not fully understood.

We further identified that MALT1 regulates Th2 and Th17 cell differentiation through the NF-κB and JNK pathways (50), a mechanism closely linked to neuroinflammatory processes in CSF of AD. Single-cell sequencing studies have revealed dynamic alterations in disease-associated microglia (DAM) during AD progression (51). Moreover, MALT1-dependent cleavage of HOIL1 modulates NF-κB signaling and inflammatory activity, contributing to a “hyper-inflammatory” phenotype (52). In AD models, pharmacological inhibition of MALT1 has been shown to alleviate amyloid-*β*-induced mitochondrial dysfunction and neuroinflammation (53). Additional work is needed to dissect cell-type–specific effects of MALT1 signaling in microglia, astrocytes, and peripheral immune cells, as well as to determine whether targeting MALT1 can modulate neuroinflammation and synaptic dysfunction in a stage-dependent manner (54). Integration of multi-omics datasets and longitudinal single-cell profiling will further help elucidate whether shared genetic variants drive coordinated changes in gene expression, metabolite levels, and AD risk.

Overall, we highlight the critical role of MALT1 as an “inflammation amplifier” in AD. By driving chronic neuroinflammation, MALT1 directly or indirectly induces neuronal death and mitochondrial dysfunction, thereby exacerbating neurodegenerative changes in AD. Consequently, targeting MALT1 represents a promising strategy to disrupt the vicious cycle of inflammation in AD and potentially slow disease progression. Future studies should focus on *in vivo* interventions aimed at modulating MALT1 activity to confirm its precise causal role and therapeutic potential in AD. These investigations could provide deeper insights into AD pathophysiology and identify MALT1 as a potential therapeutic target.

However, our study has certain limitations. Firstly, the validity of the MR study might be compromised due to weak instrumental variables. To address this, we utilized independent SNPs reaching genome-wide significance levels (*p* < 5 × 10^−6^), and the F-statistic met the threshold of >10 to avoid the effect of weak instruments. Nevertheless, sensitivity analyses are still required to assess the potential influence of weak instruments and improve the robustness of causal inference. Secondly, pleiotropy occurs when genetic variants influence multiple traits beyond the exposure of interest. While several statistical approaches were employed to detect and adjust for pleiotropic effects, these methods have their own limitations. As such, the potential for residual pleiotropy cannot be fully excluded. A larger GWAS sample size is needed to identify more CSF-related SNPs in the future. Thirdly, we did not assess a direct correlation between CSF leucine/creatine and MALT1 due to the lack of matched CSF metabolomics and MALT1 measurements. Future studies with integrated CSF multi-omics (simultaneous metabolomic and MALT1 protein measurements in the same individuals) and refined functional annotation (eQTL, chromatin interaction data and colocalization analyses) will be required to directly test whether the leucine/creatine–AD relationship is mediated through MALT1-related pathways. Additionally, pleiotropy could arise when some instrumental SNPs are linked to multiple other factors. To our knowledge, this is the first study exploring the potential causal associations between CSF metabolites and AD based on genetic data, yet there is no mention of changes in CSF metabolites under different stages of AD in existing GWAS data. Moreover, as our study’s subjects are limited to Europeans, the results may not generalize to the entire population, and any conclusions should be cautiously extrapolated. Genetic heterogeneity across populations may lead to differences in allele frequencies and biological mechanisms underlying the traits studied. Future studies should aim to include data from more diverse ethnic groups to ensure that findings are broadly applicable and enhance the external validity of MR studies.

Nevertheless, while the association between CSF metabolites and AD risk is statistically significant, the causal relationship inferred from the data requires further validation. Heterogeneity across ethnicities, genetic backgrounds, and environmental contexts could influence the generalizability of the results. In addition to the biological mechanisms, potential confounding factors must be carefully considered to avoid overstating the association. Lifestyle factors such as diet, exercise, alcohol consumption, and smoking have been implicated in both CSF metabolism and AD risk. Failure to account for these confounders may lead to biased conclusions, obscuring the true nature of the relationship. Finally, while MR analysis can establish causal relationships between CSF metabolites and AD, it cannot provide the specific effect sizes of exposure on AD. Given the use of GWAS data for analysis and meta-analysis, future experiments or trials are needed to validate these results.

## Conclusion

In summary, our integrative analysis provides robust evidence that specific CSF metabolites causally influence AD risk, acting through dysregulated metabolic pathways, gene expression changes, and immune remodeling. Moreover, we developed and validated a machine learning–based predictive model with high clinical utility. These findings advance the understanding of AD pathogenesis and highlight novel biomarkers and therapeutic targets with translational potential.

## Data Availability

The original contributions presented in the study are included in the article/Supplementary material, further inquiries can be directed to the corresponding authors.

## Footnotes

1
https://biit.cs.ut.ee/gprofiler/snpense

2
https://www.uniprot.org/
