# Supplementary figures and images for: MALT1 in cerebrospinal fluid: a prognostic biomarker and potential therapeutic target in Alzheimer’s disease
Source: Front Neurol. 2026 Jan 6;16:1732729. doi: 10.3389/fneur.2025.1732729 (PMC12815869; doi:10.3389/fneur.2025.1732729)

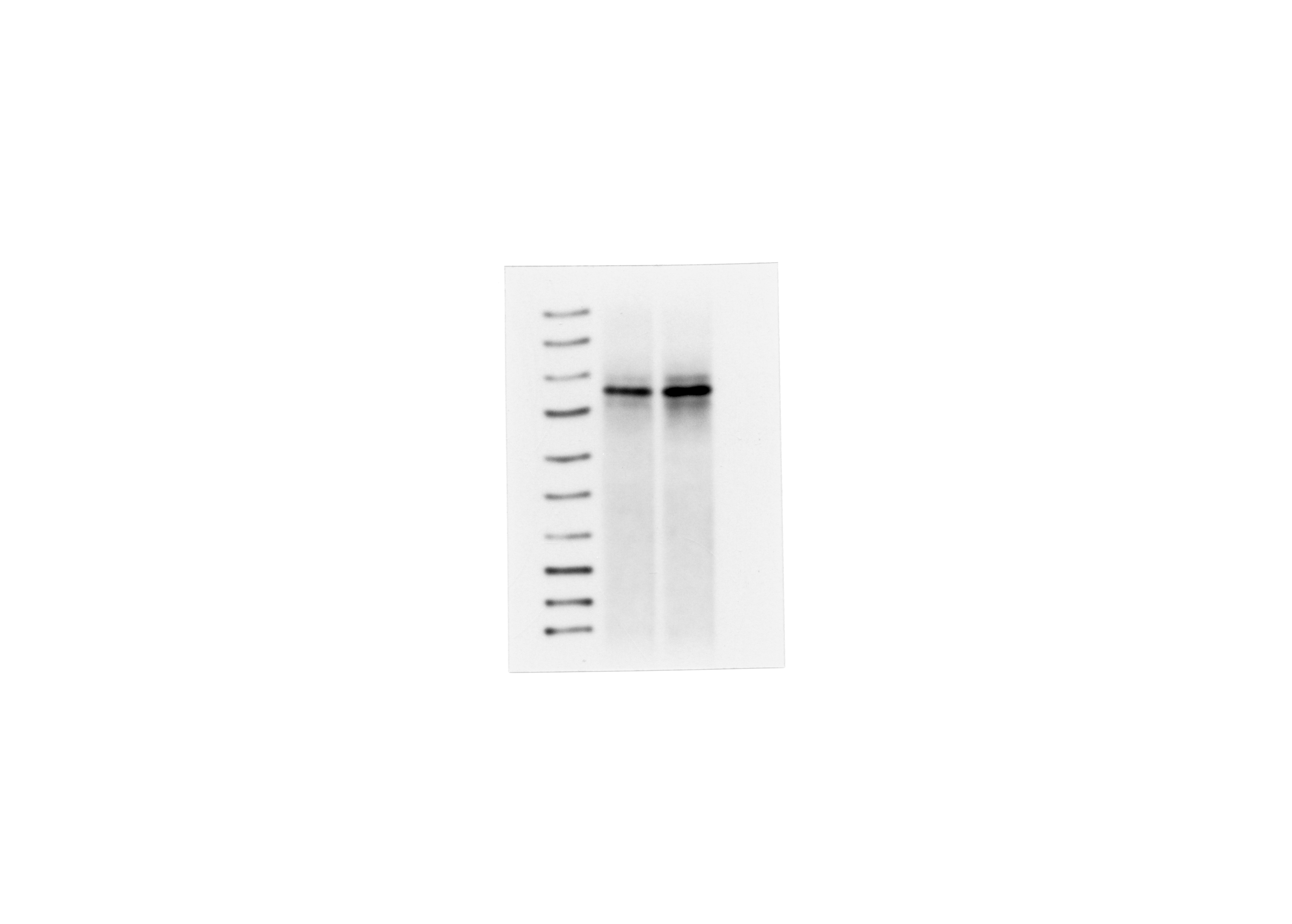

Supplement: Supplementary file 3 [file Image_1.tif]

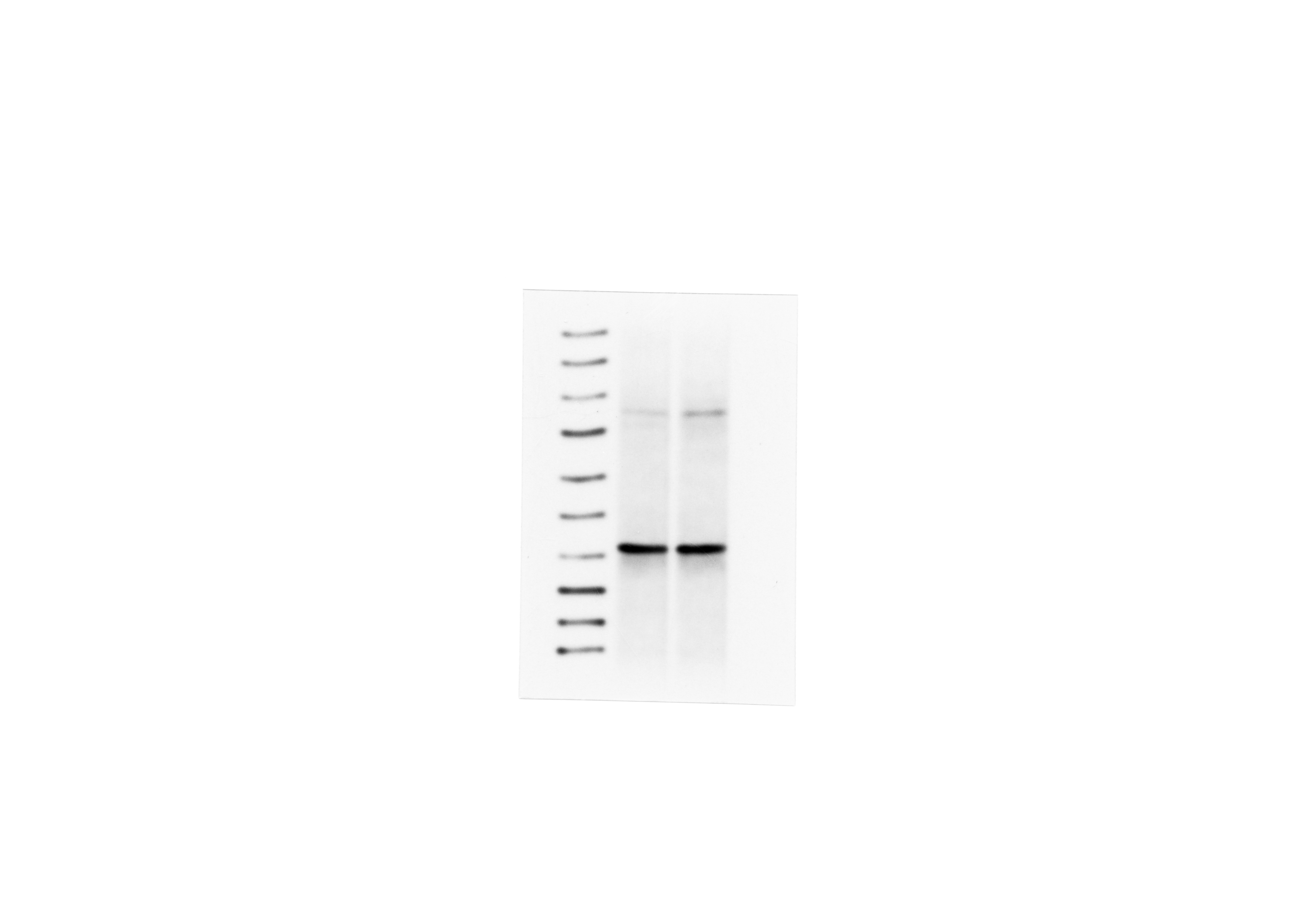

Supplement: Supplementary file 4 [file Image_2.tif]

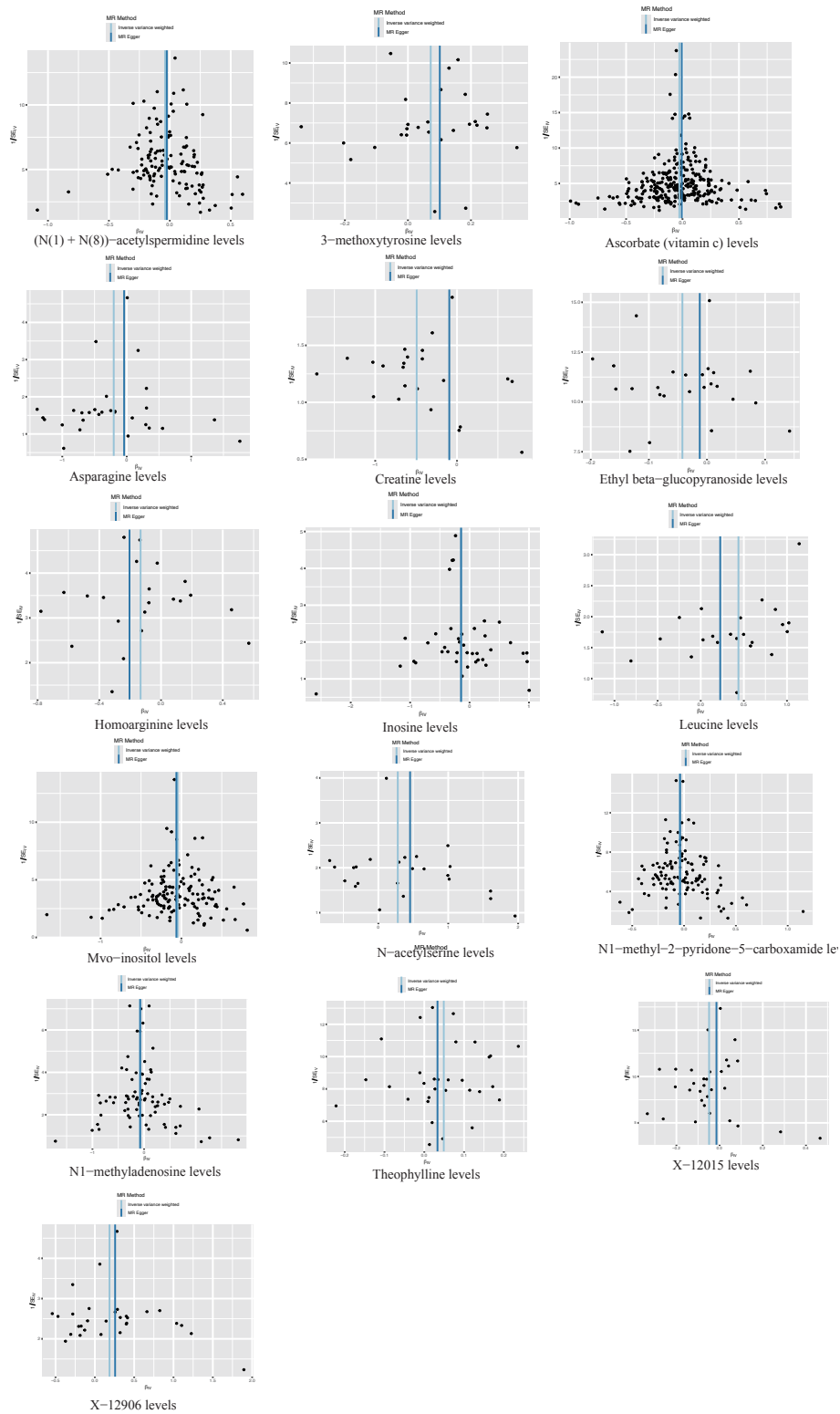

**Figure 1S Funnel plot of CSF metabolites on AD risk**

Supplement: Supplementary file 5 [file Image_3.pdf]
